# VarGenius executes cohort-level DNA-seq variant calling and annotation and allows to manage the resulting data through a PostgreSQL database

**DOI:** 10.1186/s12859-018-2532-4

**Published:** 2018-12-12

**Authors:** F. Musacchia, A. Ciolfi, M. Mutarelli, A. Bruselles, R. Castello, M. Pinelli, S. Basu, S. Banfi, G. Casari, M. Tartaglia, V. Nigro, TUDP, Raffaele Castello, Annalaura Torella, Gaia Esposito, Francesco Musacchia, Margherita Mutarelli, Gerarda Cappuccio, Michele Pinelli, Giorgia Mancano, Silvia Maitz, Nicola Brunetti-Pierri, Giancarlo Parenti, Angelo Selicorni, Sandro Banfi, Vincenzo Nigro, Giorgio Casari

**Affiliations:** 1 Telethon Institute for Genetics and Medicine, Viale Campi Flegrei, 34, 80078 Pozzuoli (Naples), Italy; 2 Genetics and Rare Diseases Research Division, Bambino Gesù Children’s Hospital, Istituto di Ricovero e Cura a Carattere Scientifico, Rome, Italy; 3 Department of Oncology and Molecular Medicine, Istituto Superiore di Sanità, Rome, Italy; 4 Università degli studi della Campania “Luigi Vanvitelli”, Caserta, Italy; 5 Department of Medical Biochemistry and Cell Biology Institue of Biomedicine, The Sahlgrenska Academy University of Gothenburg, Gothenburg, Sweden

## Abstract

**Background:**

Targeted resequencing has become the most used and cost-effective approach for identifying causative mutations of Mendelian diseases both for diagnostics and research purposes. Due to very rapid technological progress, NGS laboratories are expanding their capabilities to address the increasing number of analyses. Several open source tools are available to build a generic variant calling pipeline, but a tool able to simultaneously execute multiple analyses, organize, and categorize the samples is still missing.

**Results:**

Here we describe VarGenius, a Linux based command line software able to execute customizable pipelines for the analysis of multiple targeted resequencing data using parallel computing. VarGenius provides a database to store the output of the analysis (calling quality statistics, variant annotations, internal allelic variant frequencies) and sample information (personal data, genotypes, phenotypes). VarGenius can also perform the “joint analysis” of hundreds of samples with a single command, drastically reducing the time for the configuration and execution of the analysis.

VarGenius executes the standard pipeline of the Genome Analysis Tool-Kit (GATK) best practices (GBP) for germinal variant calling, annotates the variants using Annovar, and generates a user-friendly output displaying the results through a web page.

VarGenius has been tested on a parallel computing cluster with 52 machines with 120GB of RAM each. Under this configuration, a 50 M whole exome sequencing (WES) analysis for a family was executed in about 7 h (trio or quartet); a joint analysis of 30 WES in about 24 h and the parallel analysis of 34 single samples from a 1 M panel in about 2 h.

**Conclusions:**

We developed VarGenius, a “master” tool that faces the increasing demand of heterogeneous NGS analyses and allows maximum flexibility for downstream analyses. It paves the way to a different kind of analysis, centered on cohorts rather than on singleton. Patient and variant information are stored into the database and any output file can be accessed programmatically. VarGenius can be used for routine analyses by biomedical researchers with basic Linux skills providing additional flexibility for computational biologists to develop their own algorithms for the comparison and analysis of data.

The software is freely available at: https://github.com/frankMusacchia/VarGenius

**Electronic supplementary material:**

The online version of this article (10.1186/s12859-018-2532-4) contains supplementary material, which is available to authorized users.

## Background

Sequencing costs per megabase of DNA dropped from thousands of dollars in 2001 to the fraction of cents in 2017 [1] and hundreds of thousands of genomes are nowadays being sequenced worldwide.

High throughput sequencing (HTS) allows researchers to capture the state of human genome in space and time at an unprecedented resolution, where whole genome sequencing (WGS) and whole exome sequencing (WES) stand out as prominent techniques. Using WGS, it is possible to identify DNA variants in the complete genome of an individual while WES technology uses target enrichment kits with oligonucleotide probes that selectively pick coding regions to identify variants [2, 3]. Even though mutations in non-coding regions may be responsible for human disease [4, 5] and a consistent fraction of disease heritability remains unexplained, 85% of disease-causing mutations are found in coding regions [6], therefore WES is considered an important and very cost-effective application of HTS and has led to the discovery of the causative variants for many genetic diseases [7, 8]. Furthermore, compared to WGS, WES analysis is quicker and allows higher coverage of the coding regions [9].

With the decrease of sequencing costs, the use of HTS for diagnostics and research purpose increased exponentially resulting in high demand of parallel computing equipment for the analysis of big data. Although raw costs are definitely reduced, the data management and organization remain challenging tasks as the volume of data generated increases.

While several available resources allow to look for variants in the human genome [10–12], the availability of an internal database specific for a given project is an asset for investigating patients sharing similar signs and sympotoms, geographic location or genotype. Hence, it is imperative to develop automated pipelines able to perform routine tasks of HTS data downstream processing and mining (singletons, families or cohorts of samples) along with storage of the results in a easily readable database.

Several tools are used nowadays for the execution of pipelines for variant calling and annotation: VDAP-GUI performs analyses from FASTQ quality check to annotation [13] but is not able to build a database or automate the execution of multiple analyses. HugeSeq is a tool that can create a Variant Call Format (VCF) file (the format developed by the GA4GH consortium) starting from the FASTQ however, it has the limitation to execute only single analyses [14]. SIMPLEX is a variant discovery tool which exploits the Burrows-Wheeler Aligner (BWA) [15] and GATK [16].

It relies on the use of a cloud computing infrastructure but it does not generate a database for variant collection [17]. The tool bcbio (https://github.com/chapmanb/bcbio-nextgen) is a community effort to provide a set of pipelines for different purposes such as WES, RNA seq, miRNA seq, single cell analysis. It can analyse multiple samples but does not allow to execute multiple analyses using a single command.

Bcbio exploits the tool Gemini [18] which uses an SQLite database to store and query variants that is limited for large-scale database access. Indeed, SQLite databases allow one single write operation at any given time which allows limited throughput for further downstream analyses. Considering that the huge number of samples and sequence variants is paving the way towards more comprehensive genotype-phenotype correlation studies, being based on the SQLite database, Gemini is not suitable for implementation of new algorithms for group comparisons and sample spooling.

RUbioSeq [19] is a locally installable tool with a user-friendly Graphical User Interface (GUI) which executes pipelines for different NGS analyses but lacks the possibility to store the variant and samples genotype information into a database structure.

We previously published a paper describing a tool [20] that together with WEP [21], STORMSeq [22] and Galaxy [23] can be included in the “web tool” category that lacks fine control of analysis parameters and result compilation. Furthermore, they do not consider the local storage of data.

Taking into account all these limitations, we propose VarGenius, a software able to execute several customizable pipelines for targeted resequencing analysis (including the GBP workflow for the joint genotype analysis). It also creates a PostgreSQL database of variant and gene annotations and patient information (gender, age, kinship, phenotypes and genotypes). We designed VarGenius as a utility box for biomedical researchers with little experience in bioinformatics to run analyses with a single command as well as a tool for computational biologists to design algorithms for the discovery of mutations in cohort-level studies. The VarGenius database can be queried through SQL programming interface which has a very intuitive syntax. We provide a script (query_2_db.pl), described by the online user guide, which allows to perform basic automated queries.

## Implementation

### Variants discovery pipelines

VarGenius has been developed using PERL, while R and HTML have been used for the downstream statistics and the results web page respectively. Currently, VarGenius counts two default pipelines: one for variant analysis from WES and another for amplicon panels. Both the pipelines implement the GBP*,* which represent the most commonly used protocol for variant discovery analysis.

VarGenius does not provide a GUI. It uses, instead a straightforward Command Line Interface (CLI) based on simple PERL commands. We wrote a simple user guide (downloadable along with the software at the GitHub web page) containing a tutorial that can be used by anyone with basic Linux command line skills.

**Fig. 1:**
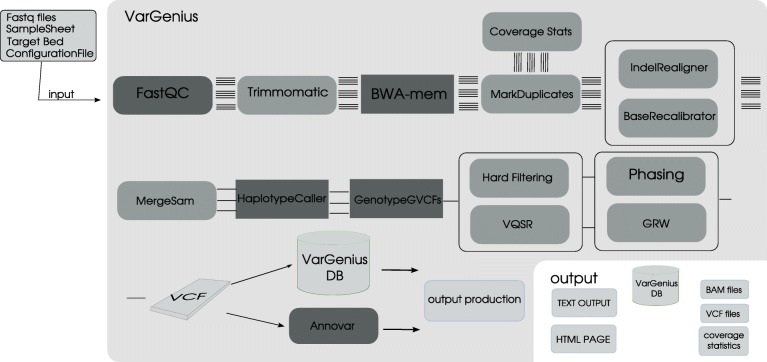
VarGenius flowchart: sequential steps allowed in VarGenius to execute different pipelines. Dark gray indicates a mandatory step, medium gray an optional one and the lighter gray represents the input and output of the pipeline. This figure also shows the input and output of VarGenius

As shown in Fig. 1, VarGenius can execute the following sequence of tasks: as a first step, FastQC checks the quality of the reads [24]. After, the user can optionally launch Trimmomatic to remove adapters and low-quality basecalls [25] (since it is not suggested in the GBP, it is not used by default in VarGenius). Mapping of reads against the reference (hg19) genome [26] is performed using BWA. Depending on the target, optional steps can be executed: duplicate removal using PICARD MarkDuplicates (http://broadinstitute.github.io/picard/), GATK IndelRealignment and GATK BaseRecalibration respectively for INDEL realignment and quality scores adjustment based on known variants. The output BAM file is used for the variant calling process by GATK HaplotypeCaller and genotype assignment with GATK GenotypeGVCFs. After the calling, SNVs and INDELs are filtered using different quality thresholds. To infer the mendelian violations in trio-based analyses and for phasing of genotypes GATK PhaseByTransmission is executed.

Whether the joint analysis of several WES samples available is preferred, it can be automatically executed by VarGenius. Additional samples can be added in further runs of the software. Variant filtering can be performed using Variant Quality Score Recalibration (VQSR). Furthermore, the Genotype Refinement Workflow (GRW) can be used to confirm the accuracy of genotype calls.

Among the VarGenius output files, we provide the VCF file which contains variant description in terms of location, genotype and quality. Once a list of variants has been called for a given analysis, they are annotated using Annovar [27], that assigns potential impact on protein function [28–30], splicing distance and the observed variant frequency in large-scale human control datasets [31, 32]. This annotation can be used to identify SNPs or INDELs that are potentially related to a known genetic disease.

Indeed, for rare diseases, one of the most useful annotation is the allelic frequency in human control datasets [33].

### Benefits of VarGenius: execution of a complete analysis with a single commands

The GBP protocol needs the manual execution of the software described above or, otherwise, requires the users to learn a scripting language (WDL) to execute the automated pipeline. Both the options could be inconvenient for users with no programming experience. One of the main benefits of VarGenius is the possibility for users to execute all the steps of the GBP protocol with a single Linux command. Figure 1 is representative of the overall process required to execute a germinal variant calling analysis. The user is asked to have the FASTQ files and the target file in BED format. Then, a sample sheet containing samples metadata and a configuration file must be produced.

Once that a sample sheet is prepared, VarGenius can be run with a single Linux command:

**perl vargenius.pl -c configuration file -ss sample_sheet.tsv – start**

This command allows the execution of all the steps of the GBP for multiple analyses. Hence, users do not have to worry about output files at each step. A single excel file will be generated containing all the variants and their annotation for all the samples included in the analysis.

### Use case: running a single WES analysis

**Fig. 2:**
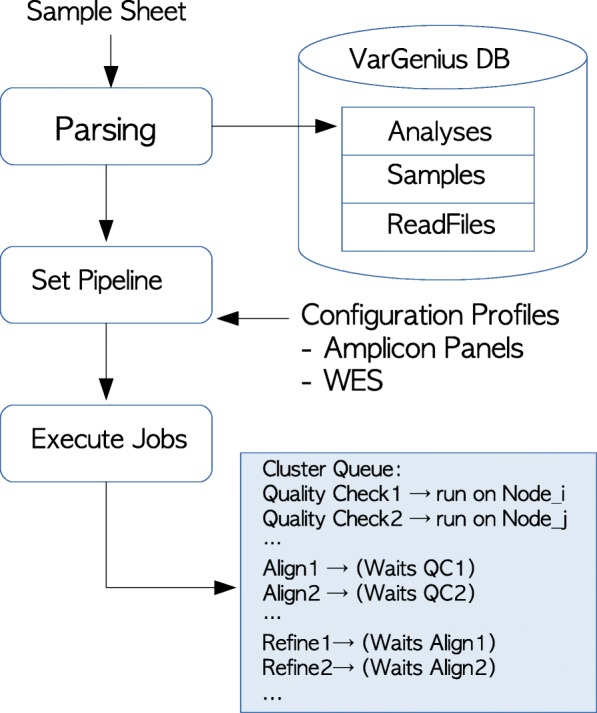
VarGenius files and data management: the samples sheet data (containing FASTQ paths, analysis, samples and read files names, sequencing type, target file and user id) is imported into the database. VarGenius automatically chooses different settings for two predefined pipelines: one for exomes and the second for amplicon panels. The different tasks (quality check, alignment, refinement, variant calling, variant filtering and output production) are executed as Torque jobs using QSUB command and scheduled in the cluster

As shown in Fig. 2, VarGenius requires a companion text file with samples and analyses information (gender, kinship, FASTQ location, target file, sequencing type) as input which we call as the *sample sheet.*

The information reported on the sample sheet is parsed and stored into the database generating a comprehensive catalog of the analyses performed useful to visualize data organization and the analyses status.

We provide a PERL script (get_sample_sheet.pl) that automatically creates the sample sheet taking in input a tab separated text file with the information about each sample.

A configuration file providing indication of the programs to be executed along with their parameters, must be also given in input. We provide it as a template with default parameters (user_config.txt).

To illustrate the simplicity of executing a VarGenius based analysis we present a practical example using FASTQ files for a family trio (proband, mother and father).

The analysis name is *AnalysisX.* For each member of the trio we have a folder containing paired-end FASTQ files from 4 lanes (a total of 8 files per sample).

Hence, three folders for the three members should be present: *FamilyX_P* (proband), *FamilyX_M* (mother), *FamilyX_F* (father) (e.g FamilyX_P will contain the following FASTQ files: FamilyX_P_L001_R1.FASTQ.gz, FamilyX_P_L001_R2.FASTQ.gz, FamilyX_P_L002_R1.FASTQ.gz, FamilyX_P_L002_R2.FASTQ.gz, FamilyX_P_L003_R1.FASTQ.gz, FamilyX_P_L003_R2.FASTQ.gz, FamilyX_P_L004_R1.FASTQ.gz, FamilyX_P_L004_R2.FASTQ.gz).

We suggest to specify the kinship of the sample into the sample name to improve the readability of the output files. For each sample, the information needed is: name, date of birth (dob), place of birth (pob), gender and if he/she is suffering from a disease (1) or not (0). The input file for the get_sample_sheet.pl script will be as following:

- FamilyX_P 09/10/2013 Naples (NA) F 1
- FamilyX_M 25/08/1975 Naples (NA) F 0
- FamilyX_F 22/02/1975 Naples (NA) M 0

Assuming that this file is named familyX_list.txt, the PERL script can be executed with the following command:

**perl get_sample_sheet.pl -dir SAMPLES/ --target_bed clinical_exome.bed --fileList familyx_list.txt --mode exome --user_id 1 --set_kinship –add_to_analysisname _TRIO --mult_lanes -o sample_sheet_familyX.tsv**

The --dir parameter specifies the folder the samples are located in; --target_bed the name of the target file; --file_list is the familyX_list.txt created before; --mode can vary from “exome” to “targeted”; --user_id is the numeric identifier for the researcher; --add_to_analysisname is a parameter useful to change the final part of all the analysis names; --mult_lanes indicates that multiple FASTQ for multiple lanes that are used for these samples; -o indicates the path to the final sample sheet.

An example of sample sheet can be downloaded as Additional file 1.

Once the sample sheet is generated it can be used in VarGenius with the following command:

**perl VarGenius/vargenius.pl -c user_config.txt -ss sample_sheet_familyX.tsv -start**

Where the “user_config.txt” is a standard configuration file which contains a default configuration of steps to execute and parameters for the programs. A tutorial in the manual guides the user through its customization depending on the system. Once that it has been customized the first time, it can be saved and used for further runs. Optionally, the parameters for the programs used by VarGenius can be modified in this file although a default configuration is already present. Different configuration files can be prepared to generate several customized configurations to use for different analyses.

### Analysis execution

A job for each task is executed: raw data quality check, mapping to a reference, refinement of the alignment, variant calling, variant filtering and output generation (Fig. 2). From quality check to variant calling, the tasks are executed in parallel for the different samples and FASTQ files (if multiple lanes are used) on multiple machines of the cluster. From genotyping to annotation VarGenius executes a unique job on a single machine for each analysis (Figs. 1 and 2). The VCF file produced by GATK and the output from Annovar are parsed and unified to generate a final tabular output containing a row for each variant with the annotation and the samples genotypes (an example output is shown in Additional file 2). Variants, genotypes and annotation of variants are stored into the internal PostgreSQL database.

### VarGenius database

**Fig. 3:**
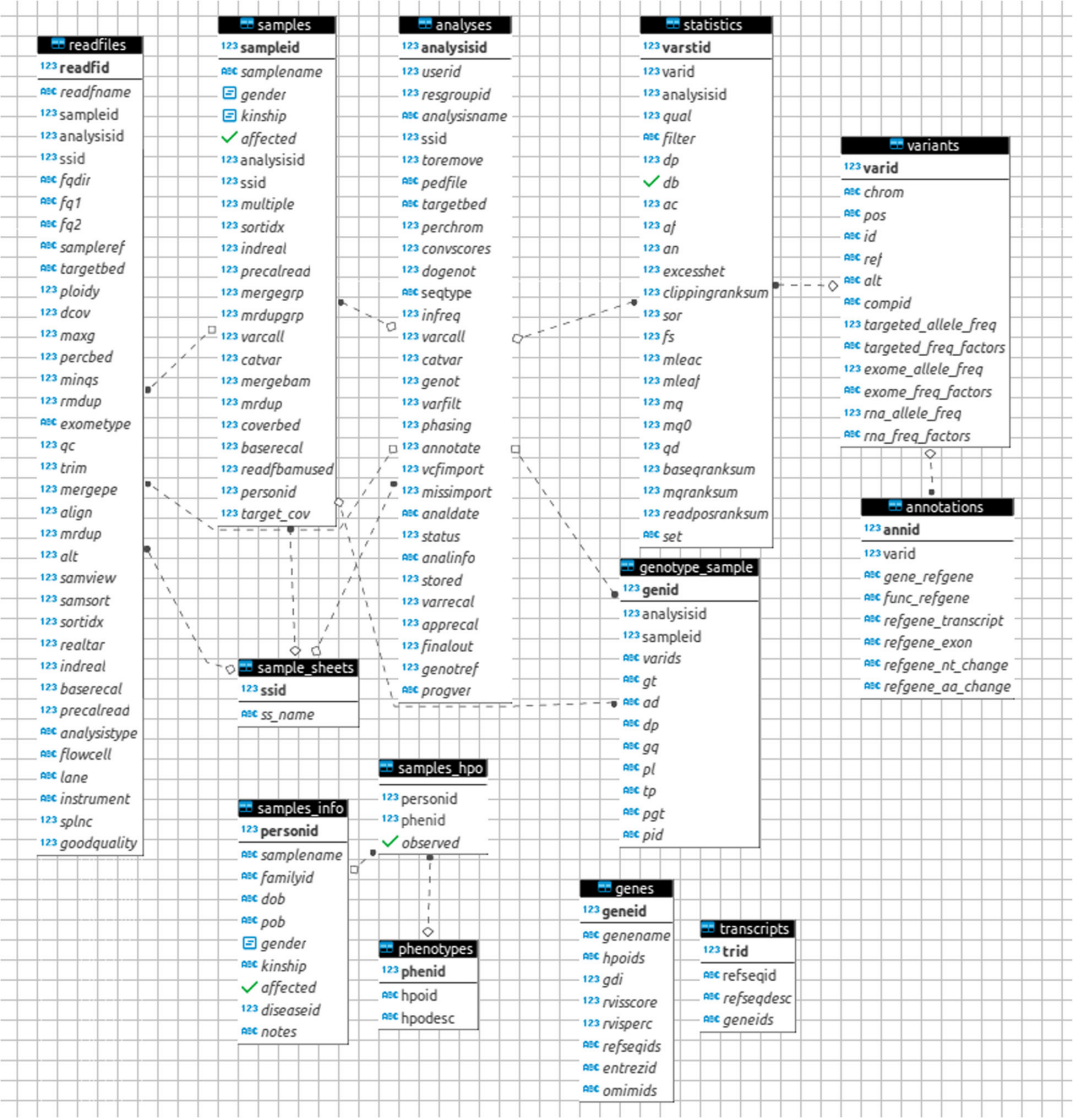
VarGenius database schema: analyzes and samples information is managed at three different levels using the *analyzes, samples and readfiles* tables. They are also used to keep track of the steps executed. The *variants* table contains the information about the variants and their allelic frequencies. The *statistics* table contains the information about the variant for each analysis (quality score, depth, etc). The *genotypes* table contains the genotype information obtained with GATK HaplotypeCaller while the *annotation* table contains more specific information about the variants calling (gene, transcript, exon, nucleotide and aminoacidic substitutions). The last three tables (*transcripts, genes* and *phenotypes)* contain the information to build the gene annotation

The database structure is displayed into an entity relationship diagram (Fig. 3) and organized in four main sub-groups: analysis management, sample information management, variant management and the external databases. The analysis management sub-group contains four tables: *readfiles*, *samples*, *analyses*, *sample_sheets*. These tables contain the localization of the files in which the reads are reported, the information about the samples and the analyses, and flags indicating that each of the steps has been successfully executed and the output is ready to undergo the next steps.

The table *sample_sheets* contains a reference to any sample sheet parsed and stored.

The sample information management sub-group includes: *samples_info*, *samples_hpo* and *genotype samples*. The samples_info table contains personal information for each sample, and the samples_hpo table contains the sample phenotypes described using Human Phenotype Ontology (HPO) identifiers. The *genotype_sample* table contains the genotype information for each sample (as detected by GATK HaplotypeCaller). The variant management sub-group of tables includes: *variants*, *statistics* and *annotations*. The *variants* table contains the chromosomal position of the variants and their allelic frequencies. Variant allelic frequencies are distinct for WES and amplicon panels. The allelic frequency is computed for all variants present into the database, generating an Internal Variant allelic Frequency (IVF). For its calculation, VarGenius retrieves from the database the number of times that the variant is found in a heterozygous state and the number of variants for which there is a homozygous alternate allele.

Only the variants for which GATK is able to compute the genotype are used for the calculation of frequencies.

It is possible to calculate the internal variant allelic frequency adjusting for relatedness or by subgroup of samples. This can be achieved by using the script query_2_db.pl (with –function FREQUENCIES) giving as input the target BED file name provided with the enrichment kit, and the user identifier or other filters embedded into an SQL command which queries the *analyses* table. The *statistics* table contains information about the variants called for each analysis (quality score, depth, etc). The *annotations* table contains additional information about the variants (gene where it is localized, transcript name, exon number, nucleotide change and, if applicable, amino acid substitution). Gene and transcript names come from the RefSeq annotation. The last three tables belong to the external databases sub-group: *genes*, *transcripts* and *phenotypes*. The *genes* table contains information about the genes reporting their association to specific HPO terms, RefSeq transcript, Entrez identifiers and OMIM genetic disorders. Furthermore, we added for each gene two scores: the Residual Variation Intolerance score [34] and the accumulated mutational damage index (gene damage index: GDI) [35].

### Web site and coverage statistics

**Fig. 4:**
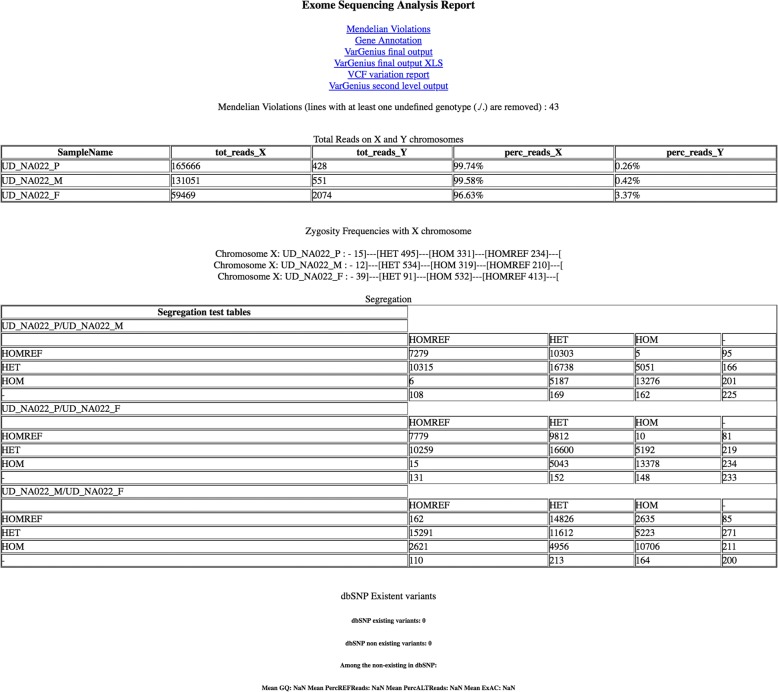
HTML page with results. This page is given as for example, it is the first page of the web site produced and shows how the results are organized. Links to download the output files and tables showing quality check statistics are present

Coverage statistics analysis is performed using GATK tools and plots are printed using ad-hoc R scripts. Summary plots and tables can be visualized into a web site where the user can find links to the generated data (FastQC quality reports, the tabular output with the annotations in text and XLS formats, BAM files for the alignments and an XML session file to visualize the BAM files in IGV (Integrative Genomics Viewer) [36]. An example of the web page is in Fig. 4.

VarGenius runs the program Samtools flagstats that generates statistics about the alignments and the removed duplicates, and the GATK DepthOfCoverage tool that computes the coverage for each sample and each gene for the entire cohort of the analysis. Low coverage gene regions are identified leveraging PICARD tools.

**Fig. 5:**
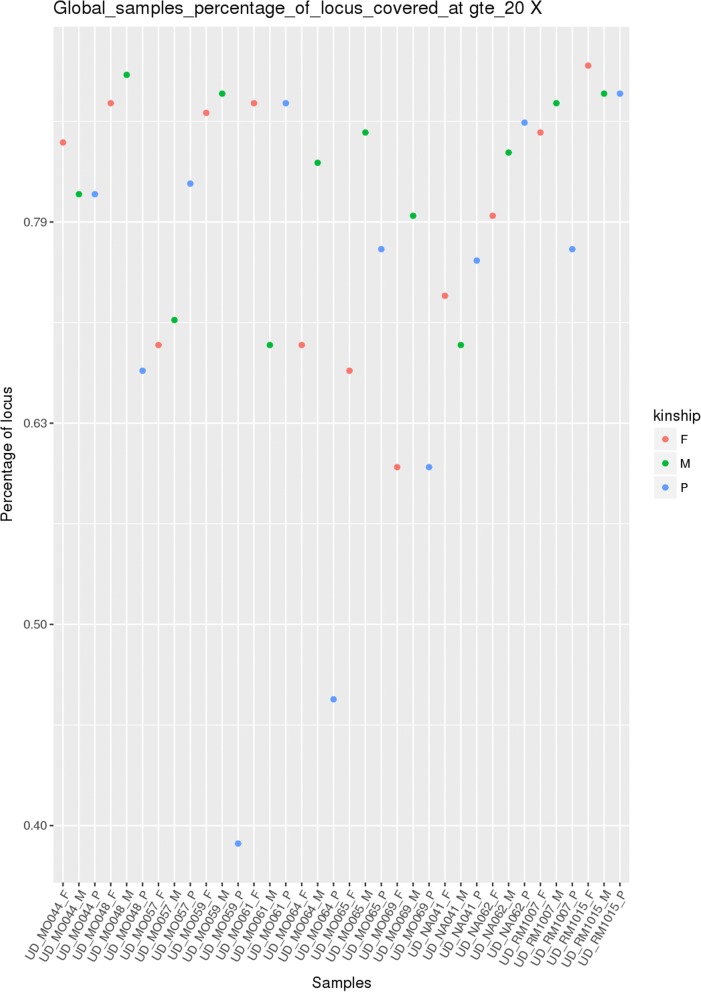
Example figure for global percentage of target coverage plot. This plot shows the percentage of the target covered by samples obtained with the same target file and can be used for sequencing run evaluation. This kind of plot is print by VarGenius at different levels of coverage (1X, 10X, 20X, 40X, 80X,100X). The figure shows the coverage of the target at 20X. Three different colors are used for kinship of the samples (probands, mothers and fathers)

VarGenius generates a plot to easily visualize the global sample coverage showing the coverage level of each sample. This plot is useful to verify the coverage of different sequencing runs performed with the same enrichment kit (Fig. 5). Further statistics are displayed in additional (not shown) images: 1. boxplots related to the coverage of genes for 4 specific diseases (they can be used to check if there are genes related to a specific disease with a coverage lower than a pre-defined threshold); 2. plots correlating the number of alternative alleles called and the genotype identified in GATK which are useful to check if the genotype inferred is correct. In the latter plot, for homozygous reference genotypes, the distribution should flatten towards the origin of the x-axis, while for the non-reference genotypes, the trend should be reversed. However, for heterozygous genotypes, the distribution should look Gaussian.

### Stop and restart method

An analysis of VarGenius is composed of six tasks: quality_check (FastQC and Trimmomatic); alignment (BWA, MarkDuplicates); refinement (BaseRecalibration); variant calling (HaplotypeCaller and GenotypeGVCFs); variant filtering (GATK VariantFiltration or VQSR pipeline); final_out (Annovar annotation, generation of the output table, statistics and web page creation). Each of the tasks can be executed independently with a specific command (--quality_check, --trimming, --alignment, --refinement, --variant_calling, --variant_filtering, --final_out), the configuration file and the identifier of the analysis. When mandatory, the previous step must be completed. As an example, if the user wants to run only the alignment of the sample with the database identifier 10 for AnalysisX she/he will use the following command:

**perl VarGenius/vargenius.pl –c user_config.txt –ra 10 –alignment**

VarGenius can either be run immediately using the BAM files created by this command, or the analysis can be continued later by running all the downstream commands (refine, variant calling, and so on).

For instance the following command allows the execution of the remaining part of the pipeline for sample 10:

**perl VarGenius/vargenius.pl –c user_config.txt –ra 10 --refinement, −-variant_calling, −-variant_filtering, −- final_out**

**Fig. 6:**
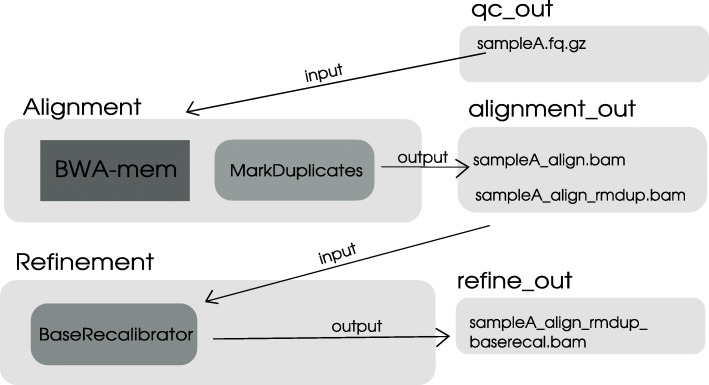
This figure details the start-and-stop method in VarGenius. At any task the input is taken from a folder belonging to the previous one. Thus, the refinement task takes the input from the alignment task and puts the output in the refine_out folder

For each analysis, VarGenius creates a folder containing a subfolder for each one of the tasks executed. As shown in Fig. 6, when the command is executed, VarGenius is in the refinement task and loads the input from the previous task (alignment_out). At the end of the refinement task, the output will be written into the refine_out folder. The next step (variant calling) will use this output as its input and so on.

This is possible because VarGenius uses flags to indicate that a specific step has been successfully executed and the output files names can be constructed by concatenating keywords linked with the flags contained into the database, (e.g. after the alignment and the duplicate removal the name of SampleA will be SampleA_align_mrdup.bam). Hence, the full name of an output file is effectively built during the execution. This design allows: 1. to stop a given analysis at any point and to restart it from the next step; 2. to choose to include or exclude steps of the pipelines; 3. to use any output file obtained with external or third-party software (e.g. BAM after the alignment or VCF file after the calling and filtering).

## Results

### VarGenius features

VarGenius has a modular backbone that makes it extremely flexible to execute several analyses using a single command. It is able to read samples and analyses information from a sample sheet and to store them into a database, so that the users have a complete set of information about the analyses executed. A single sample sheet can be used to run multiple analyses with different target files and different samples using a single command. It is also possible to add new samples to an already existent analysis making it possible to execute a “joint” analysis. When started, VarGenius creates a folder for each analysis where results and log files are saved and executes the tasks in parallel.

VCF files from different pipelines can be annotated and incorporated into the VarGenius database inserting the file in the appropriate folder. From there, it is possible to execute the annotation task.

The analysis management sub-group of database tables are used to keep track of the steps already executed. Since VarGenius builds intermediate output file names, an analysis can be stopped and restarted, in addition to being able to exclude optional steps in the pipeline. To track program execution, log files are created for each task, providing real time information on which step is being executed and displaying possible error messages.

The described features lead to an efficient management of output files and errors, making it faster to re-run an analysis. Therefore, multiple organizational bottlenecks, encountered in setting up such a pipeline are avoided. Consequently, the time saved grows linearly with the number of analyses.

### Efficient use of HPC resources

VarGenius allows customizable HPC resources request: the memory and number of CPU request can be changed according to the different tasks to be executed using the configuration file. Hence, after running few analyses, the user can adapt the resource requests to the time and memory needed by each task and reduce the queuing time for the jobs in the cluster as the jobs asking for fewer resources are automatically given priority. VarGenius also uses a background script that removes a job from the queue when the previous one is not running (i.e. interrupted for some reason) and is able to delete all the temporary files created during the analyses. This feature is crucial in the storage management because the output file that are not used for downstream analyses could rapidly result in running out of disk space.

### A local database for data management

VarGenius provides a useful database for computational biologists to implement their own SQL queries, to make cross-samples and cross-analyses searches.

We also provide a PERL script to automatically execute several queries against the database. For example, given a variant, the script returns a list of patients having that variant; given a gene, it returns all the variants present in the database located on that gene; given a list of samples and a gene name, it returns the coverage of that gene for each sample; given a list of samples, it returns the number of variants that each sample has in each gene (the list can be further filtered per deleterious, synonymous and non-synonymous); given a list of sample and HPO identifiers, it returns the list of samples having at least one among the given phenotypes; given a target file and an user identifier, it returns the allelic frequencies restricted to the resulting subgroup of samples. Hence, allelic frequencies can be categorized by cohorts.

**Fig. 7:**
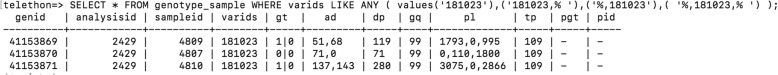
An example query to our database to identify which samples have a specific variant

As an example, using SQL syntax, the user can obtain the number of analyses where a given variant (e.g. chr19 1234606 C T) is called using what we identify as a composite identifier: i.e. a unique string containing the chromosome number, the position, the reference and alternative nucleotides concatenated with underscore (e.g. chr19_1234606_C_T):

SELECT * FROM statistics WHERE varid IN (SELECT varid FROM variants WHERE compid = 'chr19_1234606_C_T');

The variant can be also obtained using the query:

SELECT varid FROM variants WHERE compid = 'chr19_1234606_C_T' that gives in output the identifier of the variant (181023 in our database) and SELECT * FROM genotype_sample WHERE varids LIKE ANY (values ('181023'),('181023,%'),('%,181023'),('%,181023,%')); resulting in the display of all the samples in which this variant is found. Figure 7 shows the result of this query in our database: including analyses and samples where the variants are found, and the statistics correlated with the genotype called in GATK. Analyses, samples and variants are shown with their numerical database identifiers.

Using the PERL script, instead, the user has to provide a file with the list of composite variant identifiers and the command to search the variants among all the patients of the database will be:

**perl VarGenius/variants_on_gene.pl -c user_config.txt -f VARIANTS -i cand_variants.txt**

An example of the output of this command is in Table 1.

**Table 1:** Result of the query to find the variant chr6_40359875_ATGTCGAAG_A on the VarGenius database

Variant	Analysis	Sample	GT	SequencingType
chr6_40359875_ATGTCGAAG_A	FamilyX_DUO	A	0/1	WES
chr6_40359875_ATGTCGAAG_A	FamilyX_DUO	B	0/1	WES

### Challenging VarGenius with GIAB NA12878

We validated the results of the variants detection performed by VarGenius using the sample NA12878 from the Genome in a Bottle (GiaB) Consortium. We downloaded the FASTQ files with the raw sequences, the target file and a VCF file with the raw variants called on the entire genome (hg19) [37]. The FASTQ files were produced at the Garvan Institue of Medical Research (http://www.garvan.org.au/) using the Illumina HiSeq2500 sequencer and the Nextera Rapid Capture Exome and Expanded Exome enrichment kit. For the comparison we first intersected the VCF file (with calls on entire genome) with the target file and then we used the vcf-compare tool from the vcftools kit to match the two VCF files with raw calls [38]. Table 2 shows the results of the variant calling using the target file provided. The Positive Predictive Value (PPV), calculated obtaining the number of True and False Positives (PPV = TP/(TP + FP)), is of 94.6%.

**Table 2:** Results obtained using the Genome in a bottle exome sample. The column on the right shows the percentages out of the total of variants detected with the two methods

Sites Unique to VarGenius	3224 (5.4%)
Sites Unique to GIAB	1482 (2.6%)
Common Sites in VarGenius	56,164 (94.6%)
Common Sites in GIAB	56,164 (97.4%)

### Parallelization results

The implemented parallelization allows the execution of several analyses in parallel. Using an HPC cluster of 52 nodes, each one equipped with 128 GB of RAM, 2 Intel Xeon E5-2670v2 and 20 cores, VarGenius executes:

1. an entire analysis for a family (trio or quartet) in about 7 h;
2. a joint analysis of 30 WES samples in about 24 h;
3. the parallel analysis of 34 single samples from a 1 M panel in about 2 h.

The 34 FASTQ sample files were subdivided in 4 files coming from 4 different lanes of the Illumina NextSeq500 sequencing system. The average size of the FASTQ files for both read 1 and read 2 was 70 Mb.

The output file obtained by a WES trio analysis needs between 30-70GB of free space. The time needed to analyze a single sample (1 M panel) is approximately 1 h (with the same HPC cluster) and the output needs approximately 1GB of space.

VarGenius implements the Variant Quality Score Recalibration and Genotype Refinement Workflow pipelines for the joint analysis of hundreds of samples together. All the tasks for any sample are executed in parallel into the PBS cluster. The genotyping task with GATK GenotypeGVCFs is very time consuming when using several (hundreds of) samples. In order to make it faster, we parallelize the jobs running multiple GenotypeGVCFs, each one analyzing a single chromosome. The parallel execution reduces the computational time proportionally with the number of chromosomes.

### User-friendly output

VarGenius produces a tabular output, statistics, quality control files and plots, and an HTML web page showing all these results (e.g. those included in Fig. 4). The tabular output is produced both in text format and converted into XLS. An example tabular output is provided for the NA12878 WES sample in Additional file 2.

**Fig. 8:**
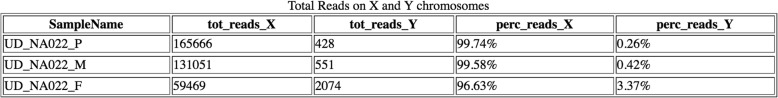
Example of a table of total reads on the X and Y chromosomes. This table is displayed in the HTML web page and shows, for each sample in an analysis, the total reads on both chromosome X and Y and their relative percentages

**Fig. 9:**
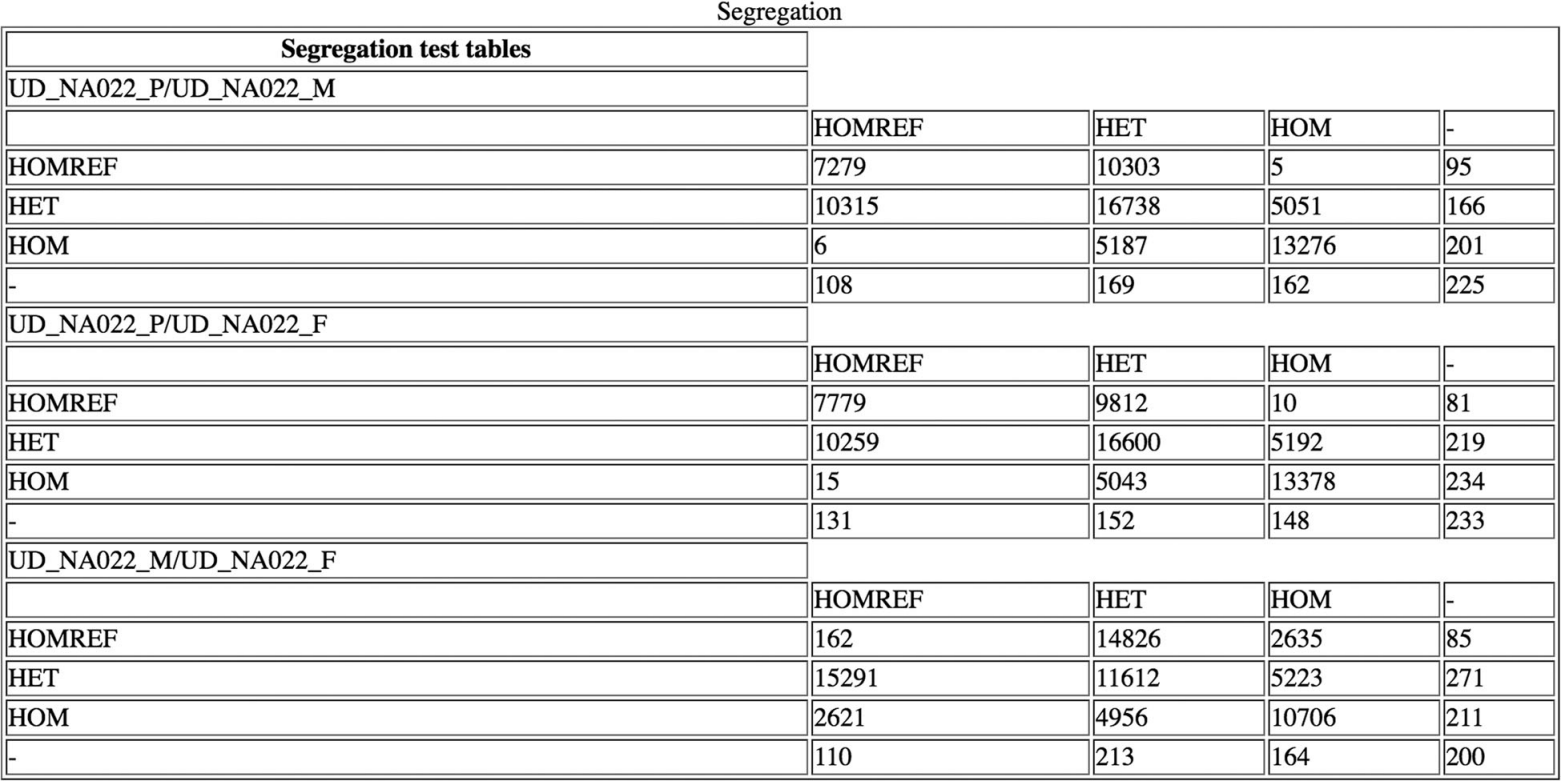
An example of segregation test table. This table is displayed in the HTML web page and matches the number of variants found in a sample with a particular genotype with all other samples. It may be used to compare calls in probands and their parents

The HTML page provides statistics useful to check the experiments. Figure 8 shows the number of reads on X and Y chromosome for an example trio while Fig. 9 shows the segregation test table that we obtain comparing the genotypes of variants in pairs of samples.

Both these tables may be useful to understand if the samples have been erroneously assigned. For instance a female will have less reads on chromosome Y compared to male.

## Conclusions

The widespread use of targeted sequencing either for research or diagnostics purposes leads to an exponential increase of heterogeneous samples that makes it necessary to develop a software able to perform multiple analyses in an acceptable time and, on the other hand, manage both the files and the outputs produced for further downstream analyses and comparisons.

We wrote VarGenius, a Linux/Unix command line software in PERL which executes the GATK best practices pipeline and can be customized using a configuration file.

This software has been tested on both WES and panels of amplicons. VarGenius provides a tabular output containing variants called and their annotations and plots and statistics shown into a website. VarGenius also implements the GATK VQSR and the GRW pipelines for the joint analysis of hundreds of samples. In this case, the tabular output contains results for all samples which can be managed using Excel or R software. The resulting variants, genotypes and annotations are loaded into a PostgreSQL database. The database can be accessed by SQL commands to visualize information about the analyses and the samples. Cross samples queries can be executed to check if a variant is present in other samples or to get its allelic frequency. VarGenius allows the user to choose the resources needed for each task. This possibility allows the jobs resources request to be adaptive with the cluster. An algorithm that takes note of the average time needed for each task depending on the dimensionality and other sample features will be also included in further development of this tool. Currently, VarGenius can be only used with a HPC infrastructure based on PBS scheduling system and using hg19 human genome reference. VarGenius has been tested using GATK3.8 and is ready to run the newer 4.0 version. We plan to include hg38 genome reference and the RNA-seq variants discovery analysis in the next release of the software.

To our knowledge, VarGenius is the first open source software for genomic variant analysis that allows the users to execute single and joint analyses of multiple samples with a single command and to create an ad hoc genomic variant database for downstream studies (samples information, variants, genotypes and phenotypes).

VarGenius is intended for investigators that have basic experience with Unix/Linux command line. Several queries and algorithms can be implemented for broad downstream analyses and population studies, making VarGenius an extremely flexible tool for genomic analyses.

VarGenius can be downloaded from GitHub where a simple tutorial guides the users through the installation and the execution.

## Availability and requirements

Project name: VarGenius

Project home page: https://github.com/frankMusacchia/VarGenius

Operating Systems: Linux systems

Programming language: PERL

Other requirements: PostgreSQL database, Perl: (tested with ver5.10), BioPerl: (tested with ver1.6), R: (tested with ver3.2.3), Java: (ver.1.8.0 or higher), BWA (ver. 0.7.15 or higher), FASTQC (ver. 0.11.3 or higher), Trimmomatic (ver. 0.36 or higher), Picard tools (ver. 2.3.0 or higher), Samtools (ver. 1.3 or higher), GATK (ver. 3.8 or higher), bedtools (ver. 2.24 or higher), Annovar (ver. 2017Jul or higher)

License: GNU General Public License (version 3 or later)

Any restriction to use by non-academics: only for research use

## Additional files

Additional file 1: An example sample sheet containing samples information that is used to start an analysis in VarGenius. (TSV 330 bytes)

Additional file 2: An example of the tabular output generated by VarGenius. (TSV 34789 kb)

## Abbreviations

CLI: Command Line Interface
GATK: Genome Analysis Tool-Kit
GBP: GATK best practices
GRW: Genotype Refinement Workflow
GUI: Graphical User Interface
HPO: Human Phenotype Ontology
HTS: High throughput sequencing
IGV: Integrative Genomic Viewer
VCF: Variant call format
VQSR: Variant Quality Score Recalibration
WES: Whole exome sequencing
WGS: Whole genome sequencing
